# Humanized Short-Stay Partial Hospitalization: Patient-Reported Outcomes (PROMs) and Experiences (PREMs) Driving Clinical and Functional Recovery in Severe Psychiatric Disorders

**DOI:** 10.1192/j.eurpsy.2026.10782

**Published:** 2026-07-31

**Authors:** A. I. De Santiago-Diaz, T. Pérez-Poo, E. Pindado-Jiménez, E. Gómez-Ruiz

**Affiliations:** 1 Psychiatry, University Hospital Valdecilla; 2 Valdecilla Research Institute-IDIVAL, Santander, Spain

## Abstract

**Introduction:**

Fighting stigma requires recognizing mental health as integral to health, ensuring dignity and respect, and involving patients and families in decisions. Patient-Reported Outcomes (PROMs) and Experiences (PREMs) enhance humanization and active participation, strengthening the therapeutic alliance. Short-Stay Partial Psychiatric Hospitalization Unit (SSPPHU-Valdecilla) is a pioneering unit for severe psychiatric disorders. By avoiding full admission, it maintains patients in their social environment while combining hospital-level intensity with community principles. Care focuses on symptom control and functional recovery through plans therapeutic integrating patient experiences.

**Objectives:**

The aim of this study is to analyse outcomes of the SSPPHU-Valdecilla intensive care model in severe acute and subacute psychiatric patients.

**Methods:**

Observational study in routine clinical practice was conducted at University Hospital Valdecilla (Santander, Spain; catchment area 300,000), approved of the local ethics committee. Adults admitted to the SSPPHU from March 1, 2020, to July 31, 2025, were included. Intensive programs include pharmacological, individual, group, occupational, and family therapies. The interdisciplinary team (psychiatry, clinical psychology, psychiatric nursing, occupational therapy) uses a transdiagnostic approach to promote clinical stabilization and functional recovery. Outcomes were assessed using standardized scales: Brief Psychiatry Rating Scale (BPRS), Montgomery-Asberg Depression Rating Scale (MADRS), Hamilton Anxiety Rating Scale (HARS), Functioning Assessment Short Test (FAST), Montreal Cognitive Assessment (MoCA), EuroQol-5 Dimension (EQ-5D), and Treatment Satisfaction Scale (CRES-4).

**Results:**

Over the past five years, 532 patients were treated at SSPPHU-Valdecilla, with a mean stay of 30 days (mean age of 47.7 years, 68% women; 42% depressive disorders, 24% psychotic disorders, 19% bipolar disorder, 15% personality disorders). Outcomes included a 75% reduction in depressive symptoms (MADRS), 70% reduction in general (BPRS) and anxiety (HARS) symptoms, 67% improvement in functional capacity (FAST), and 50% in quality of life (EQ-5D). Treatment satisfaction (CRES-4) was high: 93% satisfied (50% completely, 32% very, 11% fairly). The SSPHU-Valdecilla received two national awards: the Clinical Innovation Award from the Spanish Society of Psychiatry and Mental Health in 2024, and the 1st Prize from XI Afectivo-Efectivo Awards for humanized care in 2025.

**Conclusions:**

SSPPHU-Valdecilla provides a cost-effective alternative to full hospitalization, making treatment more accessible for patients and sustainable for healthcare systems. It promotes clinical stability and functional recovery, aiming at social and occupational reintegration, thereby contributing to healthcare efficiency and sustainability.

**Disclosure of Interest:**

None Declared

